# Diagnostic experience of intravenous leiomyomatosis with emphasis on conventional ultrasonography imaging: a single-center study

**DOI:** 10.3389/fonc.2023.1203591

**Published:** 2023-07-10

**Authors:** Zhitong Ge, Yahong Wang, Ying Wang, Wanying Li, Xiao Yang, Jianchu Li, Hongyan Wang

**Affiliations:** Department of Ultrasound, State Key Laboratory of Complex Severe and Rare Diseases, Peking Union Medical College Hospital, Chinese Academy of Medical Sciences & Peking Union Medical College, Beijing, China

**Keywords:** rare disease, gynecological tumor, intravenous leiomyomatosis (IVL), ultrasonography, ultrasonic characteristics

## Abstract

**Objective:**

Intravenous leiomyomatosis (IVL) is a rare and aggressive tumor type that has the potential to extend into the inferior vena cava (IVC) and is susceptible to be misdiagnosed and neglected. Despite its clinical significance, there is a paucity of research that has focused on the specific manifestations of IVL on ultrasonography. Therefore, this study aims to systematically analyze the specific ultrasound features of IVL and augment its diagnostic accuracy.

**Materials and method:**

Prospective inclusion was granted to patients admitted to our hospital between December 2016 and March 2021 for an IVC-occupying lesion. Multi-modal ultrasonography, encompassing gray-scale and color Doppler, was conducted. Lesions were categorized as IVL or non-IVL based on pathological or follow-up data. Two ultrasound sonographers with over 5 years of experience read and recorded ultrasound data for all lesions, which were subsequently comparatively analyzed to identify specific signs of IVL.

**Results:**

A total of 284 patients diagnosed with IVC-occupying lesions were included in the study. The lesion types comprised of IVL (n=67, 23.6%), IVC thrombus (n=135, 47.5%), tumor thrombus of renal carcinoma involving the IVC (n=35, 12.4%), tumor thrombus of liver carcinoma involving the IVC (n=24, 8.5%), leiomyosarcoma of the IVC (n=14, 4.9%), and tumor thrombus of adrenocortical adenocarcinoma (n=9, 4.1%). The presence of “sieve hole” and “multi-track” signs was observed in 20 IVL lesions under the grey-scale modality, while both signs were absent in the non-IVL group (*P*<0.01). The study found no statistically significant differences in the presentation of “sieve hole” and “multi-track” signs under the grey-scale and color Doppler modalities in cases of intravascular lithotripsy (IVL) (*P*>0.05). Using these two signs as diagnostic criteria for IVL, the sensitivity, specificity, positive predictive value (PPV), negative predictive value (NPV), miss rate, misdiagnosis rate, and accuracy were determined to be 29.9%, 100%, 100%, 82.2%, 70.1%, 0, and 83.5%, respectively (AUC ROC=0.649; 95%CI: 0.537-0.761).

**Conclusion:**

IVL exhibits distinct ultrasound presentations, including “sieve hole” and “multi-track” signs, which demonstrate high specificity and accuracy as diagnostic indicators. Furthermore, these signs are corroborated by pathological evidence and effectively distinguish IVL from other lesions occupying the IVC.

## Introduction

Intravenous leiomyomatosis (IVL) is a rare tumor with a low incidence and its prevalence rate of approximately 0.25%. The prevalence rate of IVL was ascertained by treating total 30,757 patients who underwent operation for uterine leiomyoma at Peking Union Medical College Hospital between November 2002 and January 2015 with a mere 76 individuals (0.25%) confirmed IVL by postoperative pathological results (1). Though IVL is histologically benign, it features specific malignant biological behaviors of extending into the venous lumens (2). Moreover, accurate diagnosis is challenging owing to the heterogeneous and unconventional clinical manifestations (3). Upon the emergence of symptoms, it is typically indicative of an advanced stage of IVL that has spread to the heart or pulmonary artery, leading to dyspnea, heart failure, and potentially sudden death (4). In this context, the attainment of an early and precise diagnosis of IVL is of paramount importance (5).

To date, there exists a dearth of dependable biomarkers for the preoperative detection of IVL (6). Besides, the intricate imaging manifestations of IVL frequently result in overlooked and erroneous diagnoses (7). Sun and colleagues proposed that enhancing the precise diagnosis of IVL is imperative, given its notable escalation in prevalence over recent years (8). Various imaging modalities, such as enhanced computed tomography (CT), magnetic resonance imaging (MRI), positron emission tomography/computed tomography (PET/CT), and ultrasonography, are currently employed for preoperative diagnosis of IVL; however, the optimal method for this purpose remains a topic of debate. Enhanced CT imaging has the capability to demonstrate the sponge and sieve-like morphology in partial IVL and facilitates the precise localization and comprehensive assessment of tumors. This feature holds significant importance in the fields of tumor diagnosis, surgical planning, and postoperative monitoring (9). MRI has demonstrated a noteworthy capacity to differentiate soft tissue and is a valuable diagnostic instrument for detecting IVL through visualization of the correlation between intravenous lesions and pelvic masses (10). While PET/CT has the ability to demonstrate reduced ^18^F-FDG uptake compared to other malignant disease, thereby suggesting the benign nature of the lesion; however, this modality is associated with higher costs (11). However, enhanced CT, MRI and PETCT are static images, which cannot vividly show the activity of the lesion in the blood vessel, which is of great significance for the formulation of the operation plan.

Conventional ultrasound, a widely used imaging modality that offers the distinct advantage of being radiation-free, repeatable, and capable of dynamic observation of lesions, is poised to serve as a viable alternative for IVL diagnosis due to its economic, user-friendly, and expeditious nature. Additionally, ultrasound has demonstrated efficacy in the diagnosis of various vascular-related diseases, owing to its superior tissue resolution and ability to visualize blood flow within the lesion, which should play a vital role in the detection and diagnosis of IVL.

The infrequency of IVL poses a constraint on the exploration of IVL indicators through ultrasound, thereby augmenting the intricacy of ultrasonography in the clinical diagnosis of IVL. Consequently, IVL is susceptible to being misidentified as other inferior vena cava lesions, such as thrombi or sarcomas, leading to a heightened likelihood of misdiagnosis and delayed treatment (12). Therefore, the objective of this study was to investigate specific ultrasound characteristics of IVL that possess diagnostic significance, with the ultimate goal of enhancing its diagnostic precision.

## Materials and methods

### Patients population

The present study was approved by the Ethics Committee of Peking Union Medical College Hospital in compliance with the Declaration of Helsinki and the guidelines of the Clinical Practice Coordination Conference. Prospective inclusion of patients treated for IVC space-occupying at the hospital between December 2016 and March 2021 was undertaken, with all participants providing informed consent through signed documentation. Eventually, 284 patients with IVC space-occupying lesions were identified through conventional ultrasound examination, comprising 67 IVL cases and 217 non-IVL cases.

The present study incorporated specific inclusion criteria, namely: (1) the presence of inferior vena cava space-occupying lesions; (2) prior completion of conventional ultrasonography before surgical intervention; (3) voluntary participation of eligible individuals; (4) patients undergoing surgery at our institution; and (5) age exceeding 18 years. Conversely, exclusion criteria were also implemented, which encompassed: (1) absence of preoperative clinical and imaging data; and (2) unwillingness to partake in the study.

### Examinations

Abdominal ultrasonography was conducted using a convex array probe C5-1MHz from IU22, EPIQ7, Netherlands, Philips, and a convex array probe C5-1MHz from Aplio500, i900, Japan, Canon. The procedure was performed in the morning by an ultrasound sonographer with over five years of experience, and participants were instructed to maintain a supine or lateral recumbent position. Gray-scale scanning was conducted on the abdominal venous vessels, including the inferior vena cava, bilateral renal veins, and bilateral iliac veins. Simultaneously, Doppler flow signals were obtained both around and within the lesions.

### Image assessment

**Figure 1:**
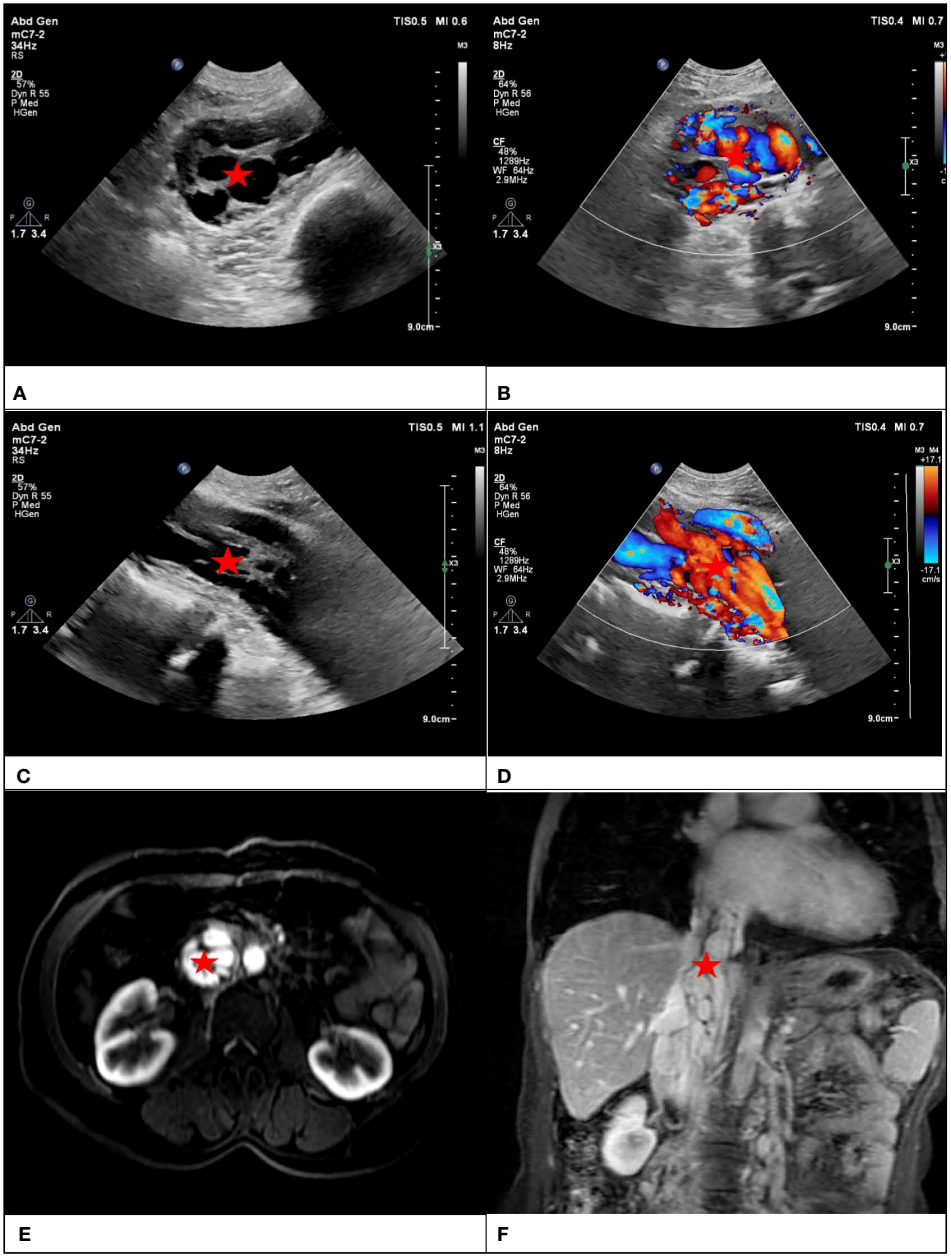
Transverse section of IVL showed the “sieve hole sign” in gray scale mode and color Doppler mode in figure **(A, B)** respectively (red star). Longitudinal section of IVL showed the “multi-track sign” in gray scale mode and color Doppler mode in figure **(C, D)** respectively (red star). Figure **(E)** showed transverse section of IVL with “sieve hole sign” and figure **(F)** showed longitudinal section with “multi-track sign” in enhanced MRI (red star).

**Figure 2:**
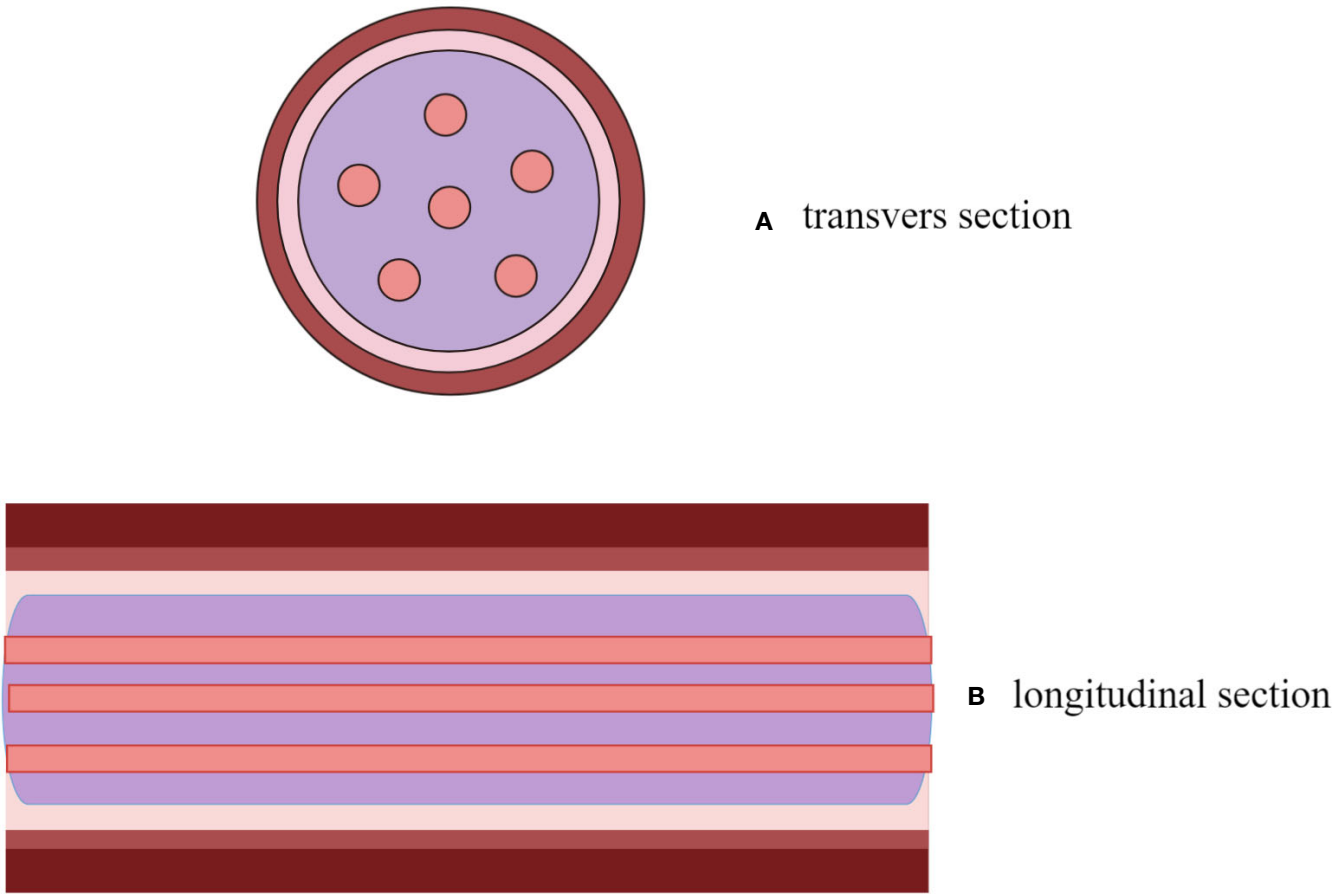
A descriptive diagram showed IVL with “sieve hole sign” in transverse section **(A)** and “multi-track sign” in longitudinal section **(B)**.

Lesion shape, echo type and homogeneity, and inside blood flow were monitored in both the transverse and longitudinal sections. To avoid inter-observer difference, signs of lesions were recorded by two ultrasound sonographers with more than 5 years of experience, and discrepancy was resolved by discussion. The two ultrasound sonographers individually listed and presented their diagnostic justifications and subsequently arrived at a consensus following a thorough exchange of perspectives. The present text provides a definition of the “sieve hole sign” and the “multi-track sign”. In grayscale mode, the lesion exhibits numerous circular anechoic spots upon transection, while a longitudinal transection reveals several parallel long strip anechoic regions, referred to as the “sieve hole sign” and the “multi-track sign” in gray-scale respectively. In color Doppler mode, blood flow signals are abundant in the circular echo and banded echo of two-dimensional gray-scale ultrasound, hence the terms “sieve hole sign” and “multi-track sign” are employed (**Figure 1**). In order to have a better understanding, the diagnostic pattern diagram is provided in **Figure 2**.

### Surgical pathology and follow-up

The pathological results of surgical resection were diagnosed by two experienced pathologists. The diagnosis of thrombosis was based on effective anticoagulation therapy, interventional treatment or surgical intervention.

### Statistical analysis

A continuous variable’s mean and standard deviation are given as a mean ± standard deviation and a categorical variable’s number is given as a percentage (%). Continuous variables were tested using the t-test or the Wilcoxon rank test. A Chi-square or Fisher exact test was performed on the count data. Interobserver agreement was assessed with a weighted Kappa test. The sensitivity, specificity, positive predictive value (PPV), negative predictive value (NPV), and accuracy of these results were calculated after comparing them with pathological results. We used SPSS (version 25.0; SPSS Inc., Chicago, IL, USA) for the statistical analysis. The area under the receiver operating characteristic curve (AUC) was determined using MedCalc (version 11.0, MedCalc, Mariakerke, Belgium). All analyses were deemed statistically significant at a level of *P* < 0.05.

## Results

### Baseline characteristics of patients

Out of the total 284 IVC-occupying lesion, 67 lesions were identified as IVL while the remaining 217 lesions were classified as non-IVL. The general and clinical data of all participants was presented in **Table 1**.

**Table 1:** General information of the cases.

	IVL (n = 67)	Non-IVL (n = 217)	*P* -value
Age (year)	46.40±6.18	51.14±16.65	*P*<0.01
Gender: (Male /Female)	(0/67)	(100/117)	*P*<0.01
Uterine surgery history	60 (89.6)	33 (15.2)	*P*<0.01
Symptoms			
Lower limb edma	12 (17.9)	81 (37.3)	
Flustered shortness of breath	11 (16.4)	8 (3.7)	
Abdominal mass	1 (1.5)	11 (5.1)	
Lumbago and back pain	8 (11.9)	2 (0.9)	
Fatigue	2 (3.0)	17 (7.9)	
Increased menstrual	4 (6.0)	1 (0.5)	
Bulge in the lower abdomen	7 (10.4)	2 (0.9)	
Syncope	3 (4..5)	0	
Chest tightness after activity	1 (1.5)	25 (11.5)	
Vaginal bleeding	3 (4.5)	1 (0.5)	
bellyache	0	39 (18.0)	
asymptomatic	15 (22.4)	19 (8.8)	
hematuria	0	11 (5.1)	
treatment			*P*<0.01
Stage I surgery	61 (91.0)	128 (59.0)	
Stage II surgery	6 (9.0)	0	
anticoagulant therapy	0	89 (41.0)	

The patient age and gender were statistically different between the IVL and non-IVL groups (*P*<0.01). The mean age at onset was (46.40 ± 6.18) years old in the IVL group and (51.14 ± 16.65) years old in the non-IVL group. All patients in the IVL group were females. Numbers of males and females in the non-IVL group were 100 (46.1%) and 117 (53.9%), respectively. Uterine leiomyoma was present previously in 60 IVL patients (89.6%) and 33 non-IVL patients (15.2%), the difference between which was remarkably significant (*P*<0.01).

Patients in both the IVL and non-IVL group showed diverse clinical symptoms. Symptoms in the IVL group were asymptomatic (incidental finding on medical examination) in 15 cases (22.4%), lower-extremity edema in 12 cases (17.9%), palpitation and shortness of breath in 11 cases (16.4%), back pain in 8 cases (11.9%) and recurrent syncope in 7 cases (10.4%). In the non-IVL group, symptoms were lower-extremity edema in 81 cases (37.3%), abdominal pain in 39 cases (19%), chest tightness after activity in 25 cases (11.5%), fatigue in 17 cases (7.9%), abdominal mass in 11 cases (5.1%) and hematuria in 11 cases (5.1%).

All patients in the IVL group underwent surgical resection, including phase I or II surgery. Patients in the non-IVL group were assigned to surgical resection (n=128, 59.0%) or anticoagulation therapy (n=89, 41.0%).

### Pathological result

Pathological diagnosis revealed that out of the total number of patients, 67 (23.6%) were diagnosed with IVL while 217 (76.4%) were diagnosed with non-IVL lesions occupying the IVC. The comprehensive pathological findings are presented in **Table 2**.

**Table 2:** Pathological results.

Pathology	N (%)
IVL	67 (23.6)
Non-IVL	217 (76.4)
IVC thrombosis	135 (47.5)
kidney cancer embolus	35 (12.4)
liver cancer thrombus	24 (8.5)
IVC leiomyosarcoma	14 (4.9)
Adrenocortical adenocarcinoma	9 (3.2)

### Ultrasound features of all the lesions involved in the study

The ultrasound features of all 284 IVC-occupying lesions are listed in **Table 3**. In 70.1% of the 47 cases of IVL, lesions were observed as solid casts, while in the remaining 29.9% of IVL cases and all 217 non-IVL cases, lesions were observed as hollow tubular structures. The observed difference between the two groups was statistically significant (*P*<0.01).

**Table 3:** Ultrasound image characteristics of all 284 cases with IVC mass.

	Ivl (n=67)	Non-Ivl (n=217)	*p* -Value
Shape			*p*<0.01
Solid cast	47 (70.1)	217 (100)	
Hollow tubular	20 (29.9)	0	
Continuity			*p*<0.01
Yes	67 (100)	124 (57.1)	
No	0	93 (42.9)	
Protruding IVC Wall			0.004
Yes	0	28 (12.9)	
No	67 (100)	189 (87.1)	
Echo Type			*p*<0.01
Hypo echo	44 (65.7)	193 (88.9)	
Medium echo	3 (4.4)	24 (11.1)	
Hyper echo	0	0	
Mixed echo	20 (29.9)	0	
Echo uniformity			*p*<0.01
Yes	47 (70.1)	180 (82.9)	
No	20 (29.9)	37 (17.1)	
Internal blood flow			*p*<0.01
Not detected	35 (52.2)	155 (71.4)	
Venous	20 (29.9)	0	
Artery	12 (17.9)	62 (28.6)	
Sieve hole sign			*p*<0.01
Yes	20 (29.9)	0	
No	47 (70.1)	217 (100)	
Multi-track sign			*p*<0.01
Yes	20 (29.9)	0	
No	47 (70.1)	217 (100)	
Snake head sign			*p*<0.01
Yes	37 (55.2)	2 (0.9)	
No	30 (44.8)	215 (99.1)	

All lesions within the IVL group exhibited continuity and had the potential to extend to the reproductive or iliac vein. Within the non-IVL group, 124 lesions (57.1%) demonstrated continuity and were verified as thrombi (constituting 65% of the total 191 continuous cases. Of the non-IVL group, 93 lesions (42.9%) were identified as segmental lesions, encompassing liver tumor, renal tumor, leiomyosarcoma, adrenal gland tumor, and partial inferior vena cava thrombi. A statistically significant difference was observed between the two groups (*P*<0.01).

The lesions within the IVL group exhibited a pattern of spreading exclusively within the confines of the IVC, with no discernible evidence of extrusion. In contrast, 12.9% of lesions within the non-IVL group demonstrated outward extrusion, characterized by continuous interruptions of the vessel wall. A statistically significant difference between the two groups was observed (*P*<0.01).

The echogenicity of the IVL group was observed to be hypo-intense in 44 lesions (65.7%), iso-intense in 3 lesions (4.4%), and mixed in 20 lesions (29.9%). In contrast, the non-IVL group exhibited hypo-intensity in 193 lesions (88.9%) and iso-intensity in 24 lesions (11.1%). The observed difference between the two groups was found to be statistically significant (*P*<0.01).

In terms of the blood flow within the lesions, venous and arterial blood flow signals were detected in 20 (29.9%) and 12 (17.9%) lesions in the IVL group. No blood flow signal was observed in 35 lesions (52.2%) in the IVL group while in 155 lesions (71.4%) in the non-IVL group. The difference in blood flow was remarkably significant between the two groups (*P*<0.01).

**Figure 3:**
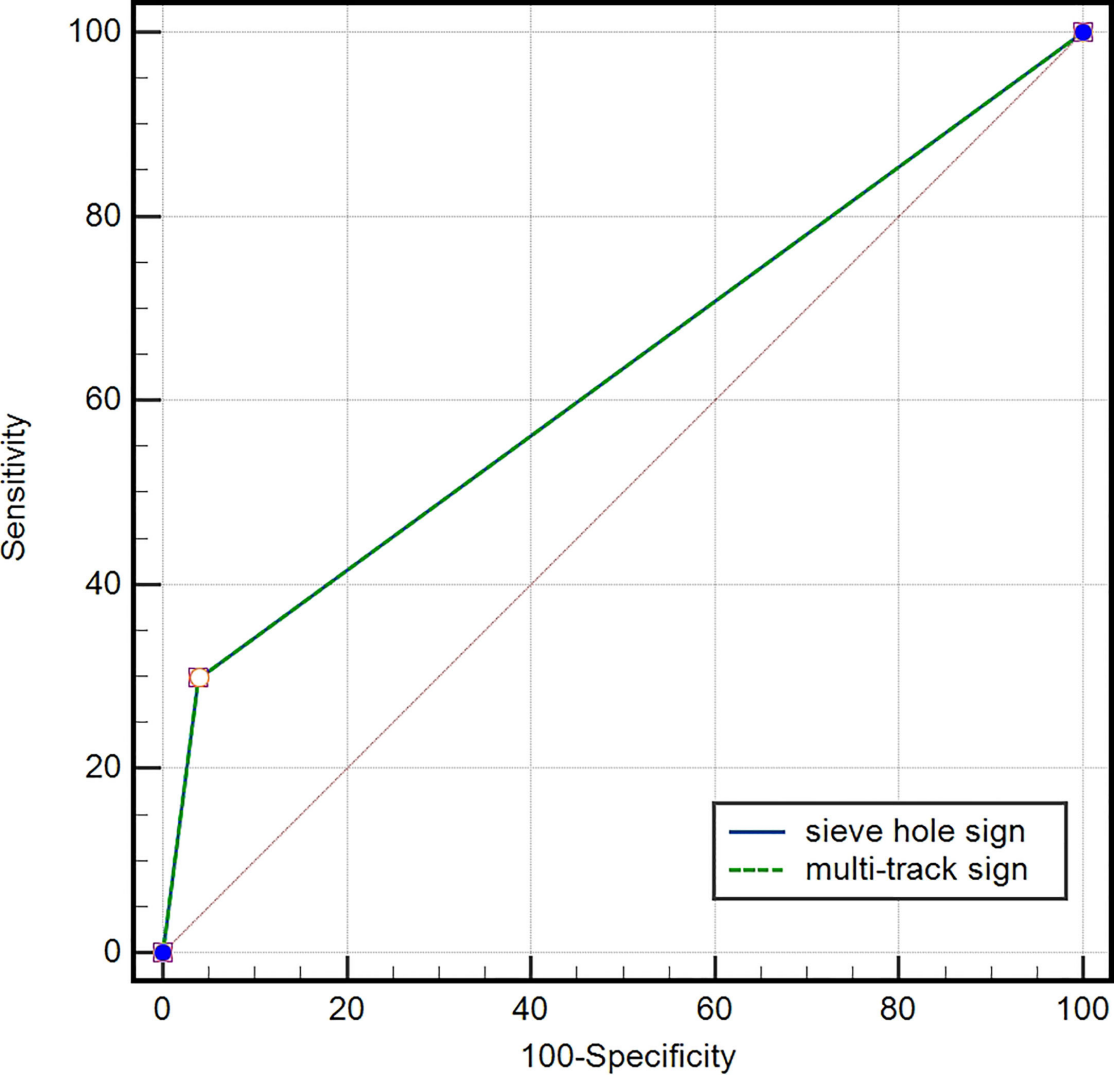
ROC curve of IVL diagnosed by “sieve hole sign” and “multi-track sign”, AUC=0.649 (95% CI: 0.537-0.761).

In the IVL group, 20 lesions (29.9%) exhibited the presence of “sieve hole” and “multi-track” signs, specifically the “sieve hole “ sign in the transverse section and the “multi-track” sign in the longitudinal section. In contrast, the non-IVL group did not display any lesions with these signs (*P*<0.01). Utilizing the “sieve hole “ and “multi-track” signs as diagnostic criteria for IVL, the corresponding sensitivity, specificity, positive predictive value (PPV), negative predictive value (NPV), miss rate, misdiagnosis rate, and accuracy were determined to be 29.9%, 100%, 100%, 82.2%, 70.1%, 0, and 83.5%,as displayed in **Figure 3** respectively (AUC ROC=0.649; 95%CI: 0.537-0.761)

### Ultrasound features of IVL with different shapes

There were no significant variations observed in the continuity and extrusion of solid cast and hollow tubular IVL lesions (both *P*>0.05). However, the echo type and homogeneity, blood flow, and presentation of “sieve hole “ and “multi-track” signs exhibited notable differences (all *P*<0.01). In particular, the 20 hollow tubular IVL lesions displayed mixed inhomogeneous echoes, predominant venous blood flow signals, and “sieve hole “ and “multi-track” signs (**Table 4**).

**Table 4:** Comparison of ultrasonic characteristics of different forms of IVL.

	Solid cast (n = 47)	Hollow tubular (n = 20)	*P* -value
Continuity			*P*>0.05
Yes	47 (100)	20 (100)	
No	0	0	
Protruding IVC wall			*P*>0.05
Yes	47 (100)	20 (100)	
No	0	0	
Echo Type			*P*<0.01
Hypo echo	47 (100)	0	
Medium echo	0	0	
Hyper echo	0	0	
Mixed echo	0	20 (100)	
Echo uniformity			*P*<0.01
Yes	47 (100)	0	
No	0	20 (100)	
Internal blood flow			0.004
Not detected	35 (74.4)	0	
Venous		20 (100)	
Artery	12 (25.6)	0	
Sieve hole sign			*P*<0.01
Yes	0	20 (100)	
No	47 (100)	0	
Multi-track sign			*P*<0.01
Yes	0	20 (100)	
No	47 (100)	0	

Furthermore, the demonstration of “sieve hole” and “multi-track” signs did not manifest any statistically noteworthy dissimilarities when assessed utilizing both grey-scale and color Doppler modals in cases of IVL (*P*>0.05). And further details were displayed in **Table 5**.

**Table 5:** Comparison of Sieve hole sign and multi-track sign in different modals.

	grey-scale	color Doppler	*P* -value
Sieve hole sign			*P*>0.05
Yes	20	20	
No	47	47	
Multi-track sign			*P*>0.05
Yes	20	20	
No	47	47	

### Ultrasound features of IVL and corresponding pathological manifestations

**Figure 4:**
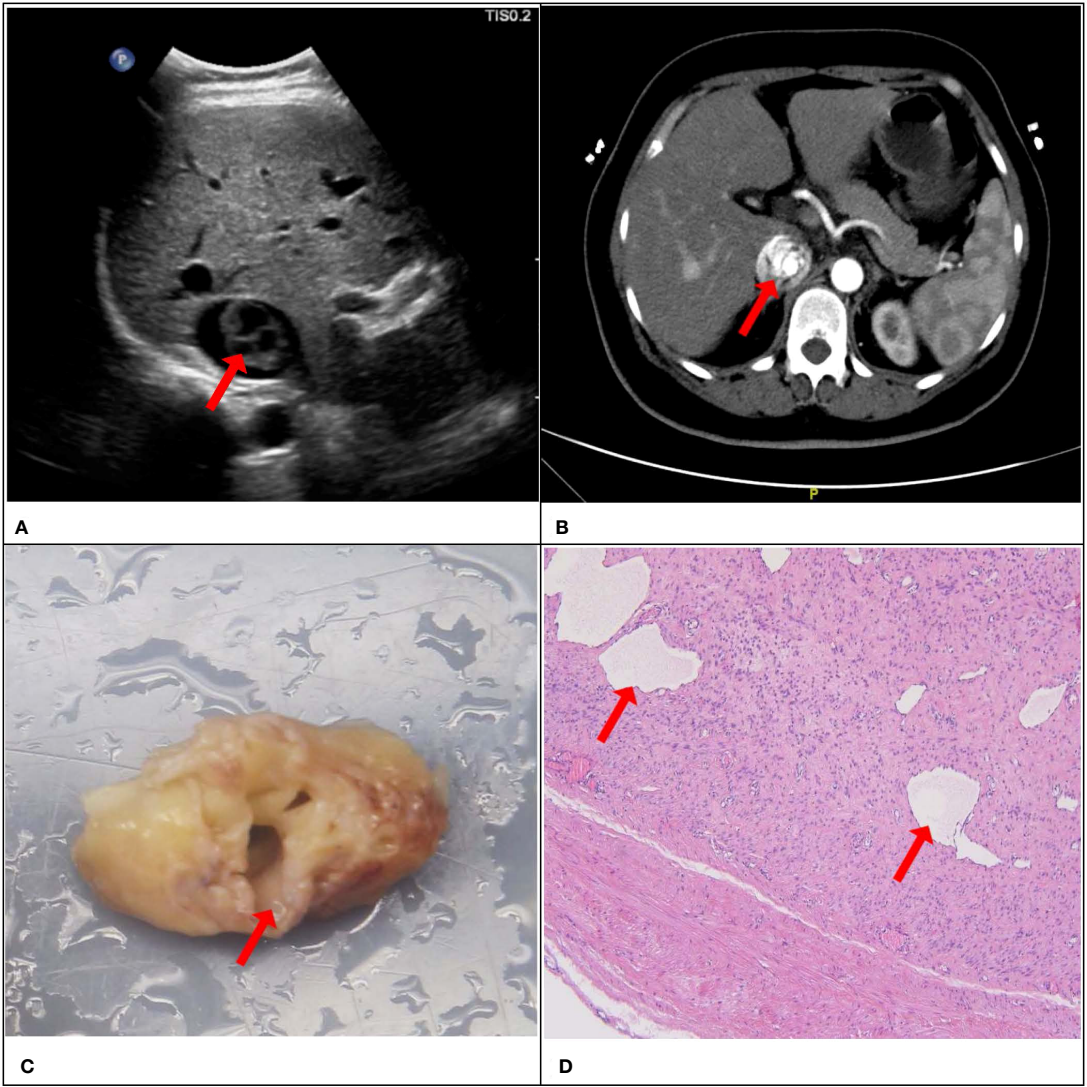
A 51-year-old female patient with a surgical pathology result of IVL. Figure **(A, B)** showed the “sieve hole sign” under conventional ultrasound and enhanced CT respectively (red arrow). The cross section in figure **(C)** showed multiple circular cavities within the gross specimen lesion (red arrow), while figure **(D)** showed the presence of small cavity structures under the microscope (red arrow).

**Figure 5:**
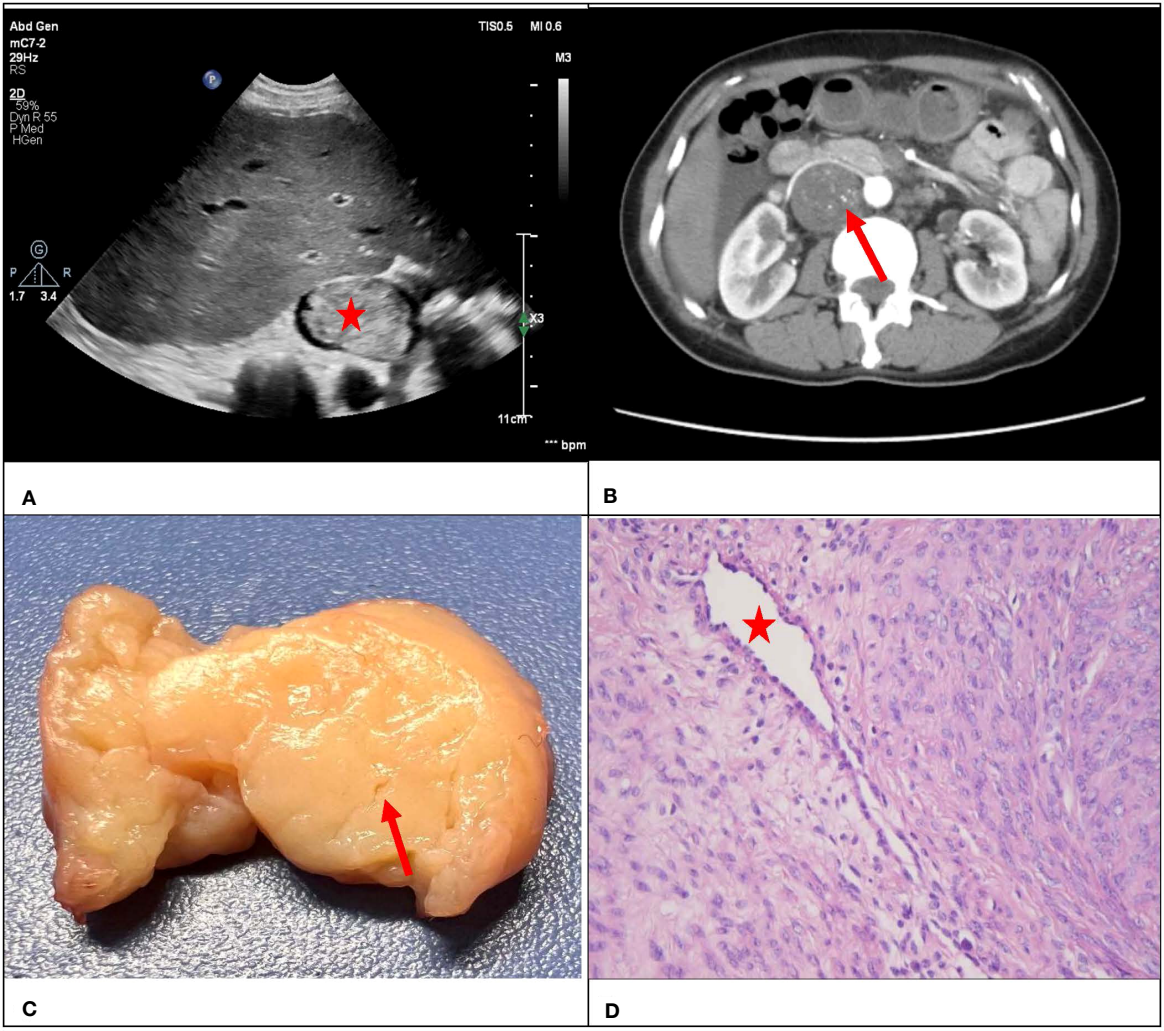
A 47-year-old female with a surgical pathology result of IVL. Figure **(A)** showed a conventional ultrasound grayscale image, with no “sieve hole sign” on the transverse section (red star). Transverse section of enhanced CT in figure **(B)** showed uneven enhancement of the lesion, presenting a small tiny “sieve hole sign”(red arrow). Figure **(C)** showed a fissure like appearance in the transverse section of the lesion (red arrow), while figure **(D)** showed the internal cavity structure of the lesion under a microscope (red star).

Consistency was observed between the ultrasound presentations and the pathological features in the 67 IVLs. The transverse section of the 20 tubular lesions exhibited a hollow tubular structure with a cystic appearance (**Figure 4**), whereas the solid cast lesions displayed a tiny lumen-like structure (**Figure 5**). Histopathologically, all lesions demonstrated spindle cells arranged in bundles, reduced karyokinesis, and the presence of lumens of small blood vessels.

## Discussion

Intravenous leiomyomatosis, a distinct form of gynecological neoplasm, is typically limited to or parasitic to the uterus, yet has the potential to infiltrate blood vessels, traversing through the uterine or ovarian veins, iliac veins, and inferior vena cava, ultimately culminating in the right atrium and potentially the pulmonary artery (13, 14). IVL was initially characterized as a neoplasm with the ability to infiltrate the venous system by Birch-Hirschfeld in 1896 (15), and subsequently in 1907 Durck reported the first case of IVL extending into the inferior vena cava and the heart, contributing to the body of knowledge on the subject (16). IVL predominantly occurs in pre-menopausal women or women of childbearing age, typically around 50 years old, and have a medical history of hysterectomy (17). A retrospective analysis revealed that a majority of patients with IVL, specifically around 64%, had undergone hysterectomy prior to diagnosis, with some patients experiencing an interval of up to two decades (18). Our study revealed that the IVL group had a mean age of 46.40 years, and a significant proportion of IVL patients, approximately 90 percent, had undergone hysteromyomatosis, which is in line with prior investigations.

The findings of our study validate the diagnostic efficacy of CU in the context of IVL. Prior research has demonstrated that enhanced CT and MRI are capable of revealing the extent and trajectory of IVL (19, 20). In our study, it was found that ultrasound has the same advantages in displaying the range and pathway of IVL lesions compared to enhanced CT and MRI. In addition, compared with contrast-enhanced CT and MRI, dynamic observation of the adhesion between intravascular lesions and vascular wall is another vital advantage of ultrasound examination in our study, which is of great significance for surgeons to make surgical plans for patients. “Sieve” and “luffa sponge” signs have been reported in enhanced CT presentation in some type of IVL, which can be viewed as a specific sign of IVL (9). And importantly, we also found the presence of this sign through conventional ultrasound. Although the proportion of conventional ultrasound found this sign is slightly lower than that of other enhanced images, it has the advantages of noninvasive, convenient and fast, without the risk of contrast agent allergy and is more economical.

There is a prevailing belief that IVL arises from the smooth muscle present in the myometrium or vessel wall, and subsequently progresses into the vein lumen (21). The manifestation of clinical symptoms in IVL is contingent upon the size of the lesion. During the initial phase, a significant number of patients remain asymptomatic, and the detection of tumors may occur incidentally (22). The findings of our study indicate that a significant proportion of patients with IVL, specifically 22.4%, were asymptomatic, thereby posing a challenge to the accurate diagnosis of the condition. As the disease progresses, various symptoms may manifest in patients, including but not limited to lower limb edema, abdominal or pelvic pain, or discomfort (23, 24). Only 17.9% of IVL patients in this study had lower limb edema. Thus, preoperative diagnosis of IVL is always tricky due to its diverse and atypical clinical manifestations (25). If a patient has a uterine tumor, accompanied by lower limb swelling, chest tightness, chest pain, congestive heart failure, and even syncope, the extension of IVL to the inferior vena cava and heart should be suspected (26, 27).

IVL lesions typically exhibit continuity, originating from either the internal iliac vein or the reproductive vein, and may subsequently extend upwards into the inferior vena cava before ultimately reaching the heart (28, 29),which was also exhibited in our study. Localized lesions such as sarcoma, liver tumor, and renal tumor, which are not treated with intravascular therapy, have the potential to extend along the venous blood return pathway (30). A report was also documented regarding IVL that exhibited segmental growth within the inferior vena cava and posed difficulty in differentiation from intraluminal IVC sarcoma (31). In our study, all IVL lesions within the venous lumens were observed to be continuous in nature and could be effectively distinguished from segmental lesions, such as sarcoma and tumor thrombus.

Currently, there is no definitive method for the diagnosis of IVL. However, many imaging techniques, including ultrasound, magnetic resonance imaging, and computed tomography, have been taken to diagnose IVL. Enhanced CT/MRI was superior in observing the lesion range and extension route. A study comprising of nine patients diagnosed with IVL revealed that enhanced CT offers distinctive benefits in the diagnosis of IVL (9). Firstly, enhanced CT can directly exhibit the tumor’s location and extension path, which is advantageous for devising an operation plan. Furthermore, IVL exhibited heterogeneous enhancement on enhanced CT, and the sponge-like and sieve-like manifestations of luffa sponge are useful for differential diagnosis. A further investigation comprising of 14 instances of IVL revealed that the solid mass type exhibited a quicker perfusion in contrast-enhanced ultrasound compared to the catheter type, with a lower perfusion intensity (32). This observation proved to be useful in identifying the origin and pathway of IVL, which was in line with the research conducted by Luo and his colleges (33). In contrast to conventional ultrasonography, enhanced CT and MRI require a longer duration and pose a risk of contrast agent allergy. In fact, enhanced CT or MRI is not the primary diagnostic tool for IVL, thus limiting its early detection value. Similarly, contrast-enhanced ultrasound is utilized following the identification of suspicious IVL lesions *via* conventional ultrasound, furthermore it is less commonly used and the introduction of contrast agents is relatively invasive.

Conventional ultrasound, as we all know, is a widely employed medical imaging technique that offers several advantages. Firstly, it is a non-invasive and non-destructive procedure that does not employ radioactive substances or produce radiation, thereby posing no risks or side effects. Secondly, it is a cost-effective and user-friendly method that yields immediate results. Thirdly, it has found extensive application in clinical diagnosis, including fetal monitoring during pregnancy, growth and development assessment in children, and general health check-ups. To summarize, conventional ultrasound imaging technology is a dependable, secure, non-invasive, and non-radiological approach that has emerged as a fundamental and widely utilized diagnostic imaging technique in contemporary clinical practice. Furthermore, it is able for conventional ultrasound to exhibit the morphology and spectrum of IVL, thereby aiding in the assessment of intravascular and cardiac lesions. However, the absence of specific ultrasound manifestations to IVL often results in misdiagnosis.

Here, we comparatively analyzed the ultrasound manifestations of IVL and non-IVL lesions and found a significant difference in shape (*P*<0.01), which was mainly due to the hollow tubular morphology of the IVL lesions. In addition, the hollow tubular morphology was also the main cause of the differences regarding the echo type and homogeneity, blood flow signal, “sieve hole “ sign and “multi-track” sign between the IVL and non-IVL groups. Based on these findings, we believed the hollow tubular morphology is a specific sign of IVL and corresponding ultrasound manifestations can be used as a unique diagnostic marker for hollow tubular IVL lesions. More comprehensively, the “sieve hole” sign and “multi-track” sign are highly characteristic representing various manifestations of hollow tubular lesions on ultrasound and can be also used as a diagnostic marker for hollow tubular IVL lesions. Traditional color Doppler modalities are unable to detect the micro vascularity within IVL lesions, resulting in a lack of statistically significant differences in the presentation of “sieve hole” and “multi-track” signs under both grey-scale and color Doppler modalities in IVL cases. Fortunately, the emergence of novel ultrasound techniques, including contrast-enhanced ultrasound and super microvascular imaging, may yield superior outcomes by enabling visualization of the minute blood vessels within the lesion, as evidenced by prior research (32).

The present study noted that the “sieve hole” sign and “multi-track” sign could effectively distinguish between IVL lesions and non-IVL lesions with a high accuracy. Given to solid cast IVL lesions, continuity was initially evaluated followed by differentiation from segmental IVC-occupying lesions, for tumor thrombus and leiomyosarcoma that are unable to extend to the iliac vein or the reproductive vein. No extrusion outwards happened in all IVLs. Additionally, IVL could be diagnosed upon detection of arterial blood flow signal within the lesion. However, there were 35 (74.4%) solid cast IVL lesions without any blood flow signal detected in the current study, making them challenging to be distinguished from thrombi. Therefore, we postulated that the possible cause of this phenomenon could be attributed to the profound positioning of the lesion in the inferior vena cava and the substantial abdominal wall, or the sluggish microcirculation within the lesion.

Overall, our study has substantiated the practical utility of conventional ultrasonography in the diagnosis of IVL, as it enables the precise visualization of lesion size, morphology, extent of involvement, and extended pathways by skilled clinicians. More importantly, we also proved that the “sieve hole” sign and “multi-track” sign have a high specificity for diagnosis of IVL and can be used as specific signs of IVL in ultrasound diagnosis. By utilizing the “sieve hole” and “multi-track” indicators, sonographers are able to efficiently and precisely diagnose hollow tubular intravascular lymphoma through the use of ultrasonography.

This study conducted a prospective analysis of the ultrasound characteristics of IVL and compared them with other lesions occupying the inferior vena cava. Additionally, the study investigated the diagnostic efficacy of the “sieve hole” and “multi-track” signs and demonstrated their specificity. Given the cost-effectiveness, ease of use, and lack of radiation associated with ultrasonography, the aforementioned signs have the potential to enhance the diagnostic precision of IVLs using this modality. In our study, the sensitivity, specificity, PPV, NPV, miss rate, misdiagnosis rate and accuracy of the “sieve hole” and “multi-track” signs for diagnosis of IVL were 29.9%, 100%, 100%, 82.2%, 70.1%, 0 and 83.5%, respectively (AUC ROC=0.649; 95%CI: 0.537-0.761). Despite the lower incidence of “sieve hole” and “multi-track” indicators observed in this investigation through conventional ultrasound in comparison to our prior study utilizing contrast-enhanced ultrasound, the former approach is more cost-effective and accessible (34). Conventional ultrasound may serve as a significant diagnostic tool for IVL due to its comparative advantages in terms of cost-effectiveness, convenience, and absence of radiation when compared to enhanced CT/MRI.

It is imperative to differentiate IVL from various ailments such as venous thrombosis, Budd-Chiari syndrome, right atrial myxoma, primary leiomyosarcoma, and endometrial stromal sarcoma (35, 36). Venous thrombosis is characterized by the absence of neovascularization and is not amenable to enhancement by CEUS, as it is associated with a dearth of nourished blood vessel proliferation (37). Budd-Chiari syndrome is characterized by the existence of lesions that occupy space in the hepatic vein and inferior vena cava segment, resulting in a state of partial or competitive vascular obstruction (38), usually accompanied by hepatosplenomegaly, severe ascites, as well as varicose veins of the thoracic and abdominal wall and lower limbs. Furthermore, right atrial myxoma exclusively impacts the right atrium, with partial infiltration of the pulmonary artery and no involvement of the inferior vena cava (39). Leiomyosarcoma is a type of malignancy that originates from the inferior vena cava. Early differentiation between primary leiomyosarcoma and intravascular leiomyomatosis (IVL) is challenging due to the presence of diverse and nonspecific symptoms (40).

The initiation of IVL is a gradual and unconscious process and is distinguished by complex clinical manifestations that are non-specific in nature. Inexplicably, most patients are still unaware of IVL even the tumor has infiltrated the inferior vena cava or beyond. Therefore, the clinical diagnosis of IVL poses significant challenges, as simple clinical symptoms are insufficient for accurate identification, necessitating reliance on relevant imaging examinations (41). Currently, the predominant approach for diagnosing and treating IVL involves surgical intervention, encompassing two stages (I and II) that are determined by the patient’s clinical presentation (42, 43). The surgical procedure entails complete excision of pelvic cavity and intravascular lesions. The estrogen-dependent nature of intravenous leiomyomatosis (IVL) suggests that bilateral ovarian resection may serve as a viable approach to mitigating recurrence rates (44). Research has indicated that the incidence of IVL recurrence can potentially reach 30% (26), thus emphasizing the criticality of postoperative monitoring. Nevertheless, there remains a dearth of efficacious pharmacological interventions that can be employed either independently or in conjunction with anti-estrogen therapy to forestall or manage postoperative disease recurrence. The obstetrics and gynecology team at Beijing Union Medical College Hospital endeavored to employ rapamycin (sirolimus) as a treatment modality for a recurrent patient who was deemed unsuitable for additional surgical intervention. Encouragingly, subsequent monitoring demonstrated a noteworthy decrease in the lesion (45).

Conventional ultrasonography possesses the ability to provide a clear representation of the shape and path of IVL as well, comparable to that of contrast-enhanced CT or MRI. Besides, the capacity to observe adhesion between the lesion and the tube wall dynamically is of noteworthy importance, which is not easily achievable through other imaging modalities. Considering its extensive application in clinical practice, combining with its convenience, rapidity, and absence of radiation exposure, ultrasound holds substantial screening potential for the identification of IVL suspicious lesions. A more precise diagnosis of IVL can be achieved through a comprehensive understanding of its specific ultrasound indicators, thereby maximizing the diagnostic value of ultrasound in IVL. And the diagram depicting the simplified diagnostic mode is available in the **Figure 2**.

Conventional ultrasound is the basis of ultrasound diagnosis. Our investigation delved into the diagnostic efficacy of conventional ultrasound in IVL and identified distinctive characteristics of conventional ultrasound in IVL (namely, “sieve hole” and “multi-track” signs) that align with the underlying pathology of IVL. The identification of lesions in the inferior vena cava that emanate from the iliac or reproductive vein and exhibit a correlation with pelvic masses warrants a strong suspicion of the presence of IVL. The existence of distinctive characteristics within this literature may facilitate a definitive diagnosis of IVL. Furthermore, the ongoing enhancement of ultrasound tissue resolution and internal microvascular flow visualization will aid in the identification of ethmoid and orbital manifestations of IVL, thereby facilitating its diagnosis and enabling a more effective differentiation from other inferior vena cava occupying pathologies. Ultimately, the investigation of correlating ultrasound characteristics of lesions with their underlying pathology will facilitate a more comprehensive comprehension of the fundamental principles of disease ultrasound imaging and enhance diagnostic precision.

Our research results, especially the discovery of the “sieve hole” and “multi-track” signs, provide strong evidence for the accurate diagnosis of IVL using conventional ultrasound. The discovery of the “sieve hole” and “multi-track” signs makes it possible for early and accurate diagnosis of IVL, allowing patients with conventional ultrasound images to avoid more complex and expensive examinations and obtain accurate diagnosis, avoid misdiagnosis, receiving the correct surgical treatment method. The promotion of the experience of this study will enable more IVL patients to obtain correct diagnosis through the simplest routine ultrasound examination, thus improving the diagnostic accuracy of IVL, a rare disease, and also highlighting the diagnostic value and role of ultrasound in IVL. Importantly, this sign is easy to recognize and master, which helps to promote experience and conduct multi center collaborative research. Based on this, it helps to discover more specific signs of IVL and continuously improve the correct diagnosis of IVL by ultrasound. In addition, in the future, with the increasing sensitivity of new ultrasound technologies, such as contrast-enhanced ultrasound and ultrasound microvascular imaging, to the display of microvascular flow within lesions, the screening and “sieve hole” and “multi-track” signs display of ultrasound IVL will continue to improve, which will help improve the accuracy of ultrasound diagnosis of IVL. For the issue of adhesion between IVL and the inner wall of blood vessels, utilizing the advantages of ultrasound dynamic observation can help to truly demonstrate the relationship between the lesion and the wall, and help solve the needs of clinical doctors for preoperative adhesion judgment.

However, our study had some limitations. Firstly, the disease under investigation is rare, resulting in a relatively small sample size due to its low incidence. Secondly, our study was conducted in a single center, the absence of multicenter data may introduce potential bias in the enrolled samples. Therefore, future research should prioritize the promotion of relevant multicenter studies to improve the quality of our finding.

## Conclusion

IVL is a continuous lesion in the venous lumen originating from the pelvic, not protruding the venous lumen, and conventional ultrasound may prove to be a valuable diagnostic tool for IVL. The specific IVL presentations as “sieve hole” and “multi-track” signs of IVL in conventional ultrasonography could be promising diagnostic signs in clinical practice.

## Data availability statement

The original contributions presented in the study are included in the article/supplementary material. Further inquiries can be directed to the corresponding authors.

## Ethics statement

The studies involving human participants were reviewed and approved by Ethics Committee of Peking Union Medical College Hospital. The patients/participants provided their written informed consent to participate in this study. Written informed consent was obtained from the individual(s) for the publication of any potentially identifiable images or data included in this article.

## Author contributions

ZG: software, data curation, formal analysis, and visualization. ZG and YaW: writing-original draft preparation and writing-review and editing. YiW, XY, CX: writing-review and editing. YiW, XY, JL: conceptualization and design, and administration and funding acquisition. All authors contributed to the article and approved the submitted version.
